# Participant observation for inquiry-based learning: a document analysis of exam papers from an internship-course for master’s students in health services research in Germany

**DOI:** 10.1186/s12909-024-05740-4

**Published:** 2024-09-23

**Authors:** Charlotte Ullrich, Sandra Ziegler, Alicia Armbruster, Michel Wensing, Nadja Klafke

**Affiliations:** 1 Medical Faculty, Heidelberg University, Heidelberg, Germany; 2 Department of General Practice and Health Services Research, Heidelberg University Hospital, Im Neuenheimer Feld 130.3, 69120 Heidelberg, Germany

**Keywords:** Inquiry-based learning, Document analysis, Participant observation, Qualitative methods, Teaching, Master’s degree program, Internship, Medical education and evaluation

## Abstract

Inquiry-based learning (IBL) is a type of problem-based learning. While IBL aims at reflecting the work of practicing researchers, only some students will pursue an academic career. We therefore designed a course that provides opportunities for IBL by applying participant observation to internship work experience inside and outside academia. Using this internship course as an example, we investigated, how master’s students in health science executed an IBL assignment regarding the application of participant observation and presentation of findings. In addition, the understanding of occupational fields provided through the IBL assignment was examined.

To determine whether learning objectives were met, a document analysis of IBL assignments comprising presentation slides and field protocols was performed. Within content analysis, a category grid was used examining (a) suitability of chosen *research objectives*, (b) execution of *field protocols*, (c) sufficiency of *reporting* and (d) extent of *reflection*.

49 IBL assignments from the years 2020–2022 were included. Sides of IBL observations were: (a) organizations providing health care (*n* = 28); (b) administrations of health care (*n* = 8); and (c) research institutes (*n* = 13). Within students’ field protocols, the level of detail of descriptions differed. 30 assignments included reflection on the methods used and research experience. In about a third of IBL assignments, indication of observation type, theoretical background and data analysis was missing.

Using participant observation of internship work-experience for IBL can serve as a teaching tool for students to develop methodological skills. For future courses, we developed a checklist to strengthen reporting, reflection and use of theory. As internships are often integrated in degree programs in health sciences similar courses could be implemented in different programs, given qualified methodological guidance.

**Supplementary Information:**

The online version contains supplementary material available at 10.1186/s12909-024-05740-4.

## Background

IBL is a type of problem-based learning in which students apply methods comparable to those of professional researchers in the respective field [1]. Across disciplines, inquiry-based learning (IBL) is considered an effective way for students to learn through self-directed investigation. Students take an active role while teachers primarily serve as facilitators rather than traditional lecturers. As a basic principle, IBL requires a learning environment that divides the scientific process into smaller units to guide students and draw their attention to specific aspects of scientific thinking. Depending on intended learning outcomes, students’ skills and interests as well as curricula requirements, formats might vary regarding suitable topics’ suitability, comprehensiveness of the learning tasks, teachers’ guidance’ and students’ autonomy (e.g. regarding provision of questions, methods and materials) [2–4]. In higher education, IBL has been shown to be beneficial for developing students’ inquiry skills and for improving their engagement, motivation and performance [5]. In addition, it helps students to develop skills for working in complex and unpredictable environments, enhancing critical thinking, [6, 7]. However, despite the benefits of using IBL, it is still relatively uncommon in higher education [5].

While IBL aims at reflecting the work of practicing researchers, only some students in health-related courses will pursue an academic career. Work experience, in contrast, aims at applying academic skills to real world work settings offering students insights for the remaining studies and preparing them for the job market after graduation. Participant observation as an ethnographic method based on field work can serve as a method providing both: (a) an opportunity to apply research methods and (b) establish an in-depth understanding of occupational fields. Overall, within health sciences, the potential of participant observation is being increasingly discussed, stressing the benefits to investigate on actual practice itself, instead of indirect questionnaire or interview-accounts, [8, 9]. In addition, ethnographic methods can contribute to narrowing the distance between theoretical knowledge and everyday practice in health service research [10]. Traditionally associated with lengthy presence and intense participation in day-to-day-life to gain holistic insights into the habits of a collective, within health sciences ethnographic data collection is often condensed to gain insights in specific sites or situations [11, 12].

Besides general characteristics of scientific practice, learning objectives of IBL assignments based on participant observation have to address specific methodological features: Based on research literature and experience, the following learning objectives can be defined: (a) Research objectives: Participant observation is suitable for research projects seeking to understand participants’ behaviour, interactions and practices in particular situations. It requires settings where direct observations and some degree of participation are sensible. (b) Field protocols: Sound results presume field notes comprising thorough documentation with detailed accounts, separation of description and interpretation and continuing reflection. (c) Presentation of results: These aspects have to be sufficiently reported. (d) Reflection: Reflection of research experience is an inherent part of both IBL and participant observation. For these learning objectives to be met, they have to be aligned with teaching and learning activities and assessment tasks (constructive alignment) [13].

### Aim and research question

Using an internship course of a masters’ program in health services research as an example, we aimed to analyze how students executed an IBL assignment with regards to the application of participant observation and presentation of findings. In addition, we aimed to examine the understanding of occupational fields provided through the IBL assignment.

## Methods

### Study design

Reflecting the explorative nature of the research aim, a qualitative research design was chosen comprising a document analysis of exam presentation slides and corresponding field protocols. Documents created as part of an educational process can provide empirical insights into the learning experience [14]. Our analysis is based on exam papers as anonymized aggregated data. The study aims at improving the quality of teaching and learning. With enrolment, students confirm in writing, that study data can be used for administrative and evaluative purposes. In additionethics approval for this study was granted by the Ethics Committee of the Medical Faculty of Heidelberg, Germany (S012/2016). Methods were reported according to the Checklist for the use and reporting of document analysis in health professions education research (CARDA) [14].

### Data collection

All IBL assignments handed in by students after 2020 were eligible for inclusion, as the curriculum was modified in 2019. Initially collected within the study program for quality management purposes, all data were electronically filed and stored on secure servers at the Department of General Practice and Health Services Research, Heidelberg University Hospital, Germany.

### Data Analysis

Data analysis focused on content using a category grid reflecting learning objectives and general internship characteristics. This deductive approach of allocating data to pre-identified themes was complemented by inductively adding sub-themes throughout data analysis. To determine whether learning objectives were met, we examined; (a) suitability of chosen *research objectives* in terms of observable (inter)actions, (b) execution of *field protocols* in terms of detail and separation of description and analysis, (c) sufficiency of *presentation* in terms of reporting as well as (d) extent of *reflection* in terms of internship and research experience. Data were analyzed by three researchers (AA, NK, CU), with prior experience in qualitative methods. Derived themes were discussed and consented regularly within the research team (AA, NK, CU, SZ). Extracted data were analyzed and reported as absolute frequencies. The software package MAXQDA, Analytics Pro 2020 was used for data management and to facilitate coding. No automation or AI tools were used.

## Approach and course concept

The IBL course, which is offered each year and was first taught in 2016, was designed for a master’s degree program in health services research and implementation science at Heidelberg University, Germany. The consecutive master’s program comprises two academic years, corresponding to 120 ECTS credit points (CP), which are equivalent to 3600 h of total student workload. The goal of the master’s degree is to train students at an advanced level of health sciences including empirical research methods. A bachelor’s degree from within health science and a proof of basic skills (180 h/6 CP) in research methods are required for entering the master’s program. However, at the time of entering the program, skills in qualitative research varied and were almost exclusively limited to qualitative interviews. The masters’ program accepts 20 students per year. About 1/3 of the students are trained within a health care profession.

### Aims of the course

Objectives of the course, which was designed as both an internship accompanying seminar and IBL course, were: (a) providing an opportunity to learn research skills in using (participant) observation as a qualitative research method and (b) establishing an in-depth understanding of occupational fields in health care.

### Content of the course

The IBL course was taught by three teachers with a background in health services research and interdisciplinary anthropology (SZ), psychology (NK) and sociology (CU) respectively. Participant observations was introduced in a mandatory 90-minute class (SZ) highlighting its historical origin within anthropology, basic ideas of ethnography, characteristics and reach of participant observation. For writing up field notes towards field protocols, a three-column chart was recommended to distinguish observations and analysis, comprising a) a detailed *description* of observations, b) *analytical notes*, documenting the reactions, questions, interpretations and ideas of the researcher c) *themes/codes* derived from a first round of open coding. Prompts for fieldnotes (e.g., description of situations and participants’, communication styles) and examples for the three-column chart were provided.

### Delivery format

The IBL course comprises a 7-week-long work placement accompanied by on-going monthly complementary classes. The total course credit sums up to 12 CP (360 h) including: (a) 280 h internship (b) attending four mandatory classes including the introduction, (c) assignments of keeping a field diary and documentation of three observations and (d) exam: 10–15 min oral presentation and following discussion (see Table 1). Students were asked to meet up with one of the teachers at least once to determine a suitable research focus. The course was designed as a pass-fail course; therefore, no mark was given.

**Table 1:** Structure and content of the IBL Course, MSc Health Service Research and Implementation Science, Heidelberg University, Germany (CP = credit point, 1 CP = 30 h workload)

	Workload/Volume	
Internship	280 h	− Time: Internship equivalent to seven weeks of fulltime work (divisible into two blocks) − Placement: Within an organization where healthcare is provided (e.g. hospital), administrated (e.g. health insurance company) and/or studied (e.g. research department). Regular own employment (e.g., as physiotherapist or student assistant) that met these criteria, was accepted for half of the credit. − Autonomy: Placement self-selected by students
Classes	6 h	− Lecture: Introduction “Observation as a qualitative research method” (90 min) − Accompanying seminar: On-going monthly classes comprising usually three students oral-presentations (90 min) (3 times mandatory attendance)
Assignment during the internship	14 h	− Notes: Keeping a field diary − Field protocols: Digitalized written documentation of detailed observation of three occasions − Consultation: Individual discussion with teachers to define a research question
Exam	60 h	− Oral presentation: Presentation with slides on the project in class (10–15 min). − Suggested structure: (a) introduction of objective, (b) short description of internship setting, (c) focus/research questions and methodological considerations, (d) presentation of field protocol extracts (e) conclusion on internship experience and observation as a research method − Field protocols: Handing in of the three field protocols with the presentation − Q&A: Discussion of presentation within class (10–15 min) − Pass-fail course
Total	360 h (or 12 CP)	

## Results

### Sample and internship characteristics

In total, 49 IBL assignments from four cohorts (defined by the year students were enrolled in the master’s program) of the years 2020–2022 were included (see Table 2). This corresponds to all presentations held during that time. Within two assignments field protocols were missing. Nonetheless, these could be included in the analysis, since the presentations comprised sufficient extracts of the field protocols. Variation in numbers of course participants were related to part-time students. Presentations typically included 15 slides, field protocols on average two to four pages per observation. All presentations and field protocols were in German, the main language of instruction within the master’s program. Of the students providing the presentation, five of the 49 were male and about a third had a professional background in health care, mirroring the general characteristics of the student population. Internship length was 140 to 280 h, either fulfilled over three to eight weeks full-time or part-time over a period of several months.

**Table 2:** Overview of sample and internships characteristics

Cohort	
Cohort 3	2
Cohort 4	11
Cohort 5	17
Cohort 6	19
**Year of presentation**	
2020	10
2021	16
2022	23
**Internship type**	
Internship	40
Accreditation of regular employment	9
**Employer type**	
*Provision of health care*	*28*
Nursing homes	2
Hospitals	26
*Administration of health care*	*8*
Health insurances and professional bodies	3
Patients’ associations and charities	2
Governmental bodies	3
*Research on health care*	*13*
Universities	8
Other research institutes	5
**Main internship activities**	
Research	31
Administration and organization	8
Health care	4
Teaching and further education	5
Other/mix	1

For the IBL assignment, most students selected internship experience (*n* = 40); the remaining students (*n* = 9) selected their professional employment in health care. Work places were (a) organizations providing health care (*n* = 28), including nursing homes (*n* = 2) and hospitals (*n* = 26), of which 22 were university hospitals; (b) other organizations in health care (*n* = 8), such as government departments and health insurances and (c) research institutes (*n* = 13), mostly universities (*n* = 8). The focus of work experiences reflected these employer types to some extent: Most students were mainly involved in research activities (*n* = 31), such as literature reviews, developing questionnaires and interview guides, data analysis and overall research management. Some students (*n* = 5) were involved in academic teaching, mainly in the role of (assistant) teachers. Provision of health care (*n* = 4) included assisting nursing and patient involvement. Administrative tasks (*n* = 8) included preparation of meetings and reports, often based on literature research.

### Research objectives

A research objective was specified in all presentations, most addressed either communication in videoconferences, e.g., concerning active participation or technical difficulties (*n* = 14) or some aspect of research practice (*n* = 11), e.g., conducting interviews. Further objectives included patient care (*n* = 7), teaching (*n* = 4) and handling of Covid-19 regulations (*n* = 4) (see Table 3: section A). Observation settings were largely either virtual (*n* = 20) or face-to-face meetings (*n* = 23), with two observations of telephone conversation and four with mixed settings. Research participants varied and often included a mix of people: Researchers were observed most often (*n* = 29), followed by health care providers (*n* = 16) and patients/relatives (*n* = 11). In 35 cases, observing students included themselves in the descriptions.

**Table 3:** Topics addressed per learning objective

Learning objective	*N*	Topics addressed
A. Students’ research objective		
**A1. Observation focus**		
Communication in Video-Meetings	14	Active participation (*n* = 2); differences between online and face-to-face meetings; successful communication; behavior of participants; meaningful use; dealing with technical disruptions; passing on information, organizational mindfulness; comparison of large and small groups; moderation of online and face-to-face meetings; conversation management; project management for distributed teams; cross-team communication
Corona-regulations	4	Communication of rules to people with and without disabilities (*n* = 2), implementation in face-to-face meetings (*n* = 2)
Home-Office	3	Boundaries between work and private life, home office with children, collaboration in home-office settings
Management culture	3	implementation of flat hierarchies; role of managers regarding cooperation; implementation of motivating management behavior
Patient care	7	Interprofessional collaboration (*n* = 3), nurses’ handling of challenges, physicians’ handling of patients’ uncertainties, informed consent in clinical trials, health care providers’ reactions to monitoring feedback
Research Practice	11	*Method use* (*n* = 6): Designing online focus groups, dealing with data quality, communication in qualitative interviews, think-aloud in usability testing, recognition of patients’ perspectives in participatory research approaches, participants responses to eHealth apps *Communication and infrastructure* (*n* = 5): Communication of research needs to device companies, decision making in research partnerships, limited research infrastructure, communication and hierarchy, non-verbal communication in research meetings
Teaching	4	digital examination formats, theory-practice transfer, design of online teaching, group formation at the beginning of a study program
Other	3	internal and external communication; cooperation at a distance; dealing with conflicts
**A2. Observation characteristics**		
Observation settings	45	Face-to-face (*n* = 23), virtual/online (*n* = 20), telephone (*n* = 2)
Observation type	25	Open (*n* = 16), covert (*n* = 5), auto-ethnography (*n* = 1), mix (*n* = 3)
**B. Field protocols**		
Anonymization	49	
Description detail*	48	Direct quotes (*n* = 42), description of participants (*n* = 12), room description (*n* = 45), sketches/pictures (*n* = 4)
Structured protocols	49	Three-Columns: (a) description of observations, (b) analytical notes, (c) themes/codes (*n* = 48); Two-columns: (a) description of observations, (b) analytical notes (*n* = 1)
**C. Presentation and reporting**		
**C1. Background**		
Background (workplace)	49	Characteristic of the employer, including sector/industry, size, location, organizational structure, role within the health care system and characteristics of the team/department where the internship took place
Background (content-related)	39	Communication and use of online meetings (*n* = 16), health care (7), good research practice (*n* = 7), organization- and management (5), corona regulations (2)
Background (theoretical perspective)	15	Communication theory (*n* = 5), organizational and management theory (*n* = 5), theories of learning (2), emotional theory, behavioral change, methodology
**C2. Method of data conduction**		
Observer involvement	37	Participant (*n* = 30), non-participant (*n* = 5), mix (*n* = 2),
Observation type	25	Open (*n* = 16), covert (*n* = 5), auto-ethnography (*n* = 1), mix (*n* = 3)
Not reported	10	
**C3. Method of data analysis**		
Description without label/author	16	e.g. inductive coding, indicating the use of memos and concepts/theory
Ethnographic analysis	11	Citing Emerson et al. [15] (11) and/or Girtler [16] (4)
Other	3	Autoethnography (1), structured content analysis (1), mix (1)
Not reported	15	
C4. Discussion of internship experience*		
Content-related findings	34	General conditions, setting and factors (incl. technology, rules) (*n* = 11), factors influencing virtual and face-to-face meetings (*n* = 10), hierarchy (*n* = 4), policy measures (*n* = 3), problems, conflicts (*n* = 3), transfer of theory into practice (*n* = 2), home office of working mothers with children (*n* = 1)
Participant-related findings	19	Communication techniques (*n* = 7), coping strategies and leadership behavior (*n* = 6), conditions for successful communication or scientific exchange (*n* = 4), factors impacting on willingness for active discussion (*n* = 2)
Personal prospects	7	Exciting experience (*n* = 4), new career prospects (*n* = 2), one-sided activity (*n* = 1)
Not reported	11	Including 1 were “reflection” was only mentioned in heading
C5. Method literature cited*		
Recommended within the course*	14	2 references, all ethnography-specific
Recommended within the Master’s program*	18	9 references, 2 ethnography-specific
Others	9	9 references, 7 ethnography-specific
Not reported	27	
D. Reflection of research experience and methods*		
Field access	12	Digital meetings (*n* = 4), number of observable people (*n* = 3), home office (*n* = 3), to little acquaintance with the observed (*n* = 1), facial expressions while masks wearing (*n* = 1)
Finding a focus	5	Continuously rephrasing of the research questions (*n* = 3), separating two research questions (*n* = 1), selecting fitting situations for observations (*n* = 1)
Observer role**	23	Active participation (*n* = 9), keeping distance/“going native” (*n* = 5), observation bias (*n* = 4), number of people observed (*n* = 3), details of observation (*n* = 2), number of situations observed (*n* = 1), language barriers (*n* = 1), participants constant awareness of being observed (*n* = 1), observer being mistakenly addressed as a medical student (*n* = 1)
Participants’ consent	10	(Partially) covert observation (*n* = 6), handling of confidentiality agreements (*n* = 3), timing and frequency (*n* = 1)
Not reported	19	

Frequency per IBL assignment, *Multiple coding possible; **Multiple coding within theme possible

### Field protocols

Most protocolled observations within reports included room description (*n* = 45), most contained direct quotes (*n* = 42), some used description of persons (*n* = 12) and a few comprised sketches and/or pictures of places observed (*n* = 4) (see Table 3: section B). Overall, detail of description varied, ranging from rather abstract monosyllabic reports with little situation-specific portrayal to comprehensive, in-depth reports with lively accounts. Within all reports (*n* = 49), anonymization was used, primarily employing pseudonyms for people observed. However, the degree of anonymization differed, some omitting indicating professions, gender and employment titles and some masking identifying characteristics of employers. While anonymization did not limit documenting observation within most reports, observations were reduced to mere generic enumeration of events in a few exceptional ones. For structuring field protocols all students used the suggested columns to separate observations and analytical notes (*n* = 49). All but one also included the third column on emerging codes and themes. The level of detail within columns and accuracy of separation differed.

### Presentation and reporting

All IBL assignments included a description of the characteristics of the employer and information on the work activities (*n* = 49) (see Table 3: section C). Most presentations (*n* = 39) included literature-based background informationon communication and use of online meetings (*n* = 16), challenges in health care (*n* = 7), good research practice and scientific integrity (*n* = 7), organization and management (*n* = 5) or the impact of Covid-19 regulations (*n* = 2). Some students explicitly listed theoretical concepts used (*n* = 15), which were mostly communication or organizational theories.

Most (*n* = 39) presentations included a description of data conduction: The majority was based on participant observation (*n* = 30), often using open observation (*n* = 16). Overall, methods of data analysis were sparsely reported (*n* = 34): General description of data analysis without references to theoretical or methodological schools or authors (*n* = 16) usually shortly indicated whether themes/codes were derived inductively and/or deductively. For describing methods of data conduction and data analysis, recommended readings of the IBL course (*n* = 18) and/or the overall master’s’ course (*n* = 14) were often used. 27 did not refer to methodological literature at all.

All presentations (*n* = 49) included some kind of conclusion addressing results, reflections and/or recommendations. Most students discussed their observations on the content level (*n* = 34) providing primarily neutral descriptions. This was discernible when students were reporting on “general conditions, settings and factors” (*n* = 11) or “factors influencing virtual and face-to-face meetings” (*n* = 10). Many students addressed the meaning of their findings in relation to the observed participants (*n* = 19) and stated that certain communication strategies (*n* = 7) or coping strategies and leadership behavior (*n* = 6) could be instructive for them at future work places. Only the minority of students discussed their results on a personal level (*n* = 7), and most of them valued the internship combined with the participant observation assessment as an exciting and stimulating experience (*n* = 4) which made them aware of new career prospects (*n* = 2). Only one student reported that she experienced the internship as a “one-sided activity” (*n* = 1).

### Reflection

Reflection on the methods and research experience were part of most presentations (*n* = 30) (see Table 3: section D). Predominantly mentioned topics were observer roles, field access and participants’ consent. The first topic (*n* = 23) included problematizations of observing while participating and the risk of overidentifying with observed people’s perspectives (“going native”) as well as observer bias due to previous experience within the field. Additionally, students saw challenges in cases where they perceived that there were too many or too few people and/or interactions accessible for observation. Field access and identification of observable situations (*n* = 12) largely referred to limitations within home-office-settings and online-meetings. Reflections on informed consent (*n* = 10) addressed the extent of consent, e.g., when, and how many times the student’s own role as researcher should be thematized, whom to inform, and how to handle confidentiality agreements regarding internship content. In addition, some students reflected on difficulties in determining a research focus (*n* = 5).

## Discussion

The objective of the IBL assignment and reflection of the internship experience point to an in-depth understanding of the studied participants’ perspectives. In addressing communication and work-culture, participant observation allowed students to investigate how everyday experiences are shaped by institutional contexts. This confirms results of a study on patient shadowing as a teaching tool in premedical undergraduate education [17] and findings of a participant observations exercise within a medical students’ course on health care for refugees [18]. This highlights the potential of observations as a data collection method to understand often tacit and hidden rules that influence health care, as it is currently thematized under the term “institutional ethnography” [19, 20].

All students embraced the IBL assignment of using participant observation taking up recommended readings and suggested strategies, e.g., the three-column chart, writing a detailed, concrete description and using direct quotes for more vividness. Students addressed significant methodological topics in ethnographic research, such as finding a focus, field access, the observer role and participants’ consent. However, reporting on some methodological aspects was incomplete: Most notably, about a third of the IBL assignments lacked indication of observation type, theoretical background and strategy of data analysis. These results show parallels to findings on reporting quality in the health sciences [21, 22] and, in particular, difficulties regarding data analysis and relation to theory [23].

A meta-analysis of 72 studies suggested that adequate guidance to assist learners is essential to successful inquiry-based learning [2]. At the same time, there is a need to create a learning environment that allows the freedom to examine a topic independently [1, 5]. The discussed assignment was limited to a section of the research process, focusing on formulating an initial research question, documenting three singular observations and reporting of first findings. Students were provided an introductory course, counseling and methodological prompts and references. Completed assignments and students’ feedback suggest that scope, time frame and workload of the assignment were suitable and guidance concerning field protocols sufficient. However, guidance regarding content of the presentation should be specified, highlighting reporting, reflection and the use of theoretical knowledge. Based on these noted discrepancies with recommended research practices and teaching objectives, we developed a checklist for future sources for students as a scaffold to address these topics more explicitly (see supplementary material 1) [24].

This study was limited to one masters’ program only; however, the diverse students’ backgrounds and skills, point to transferability of results. Data of this study were limited to written assignments, wherefore, additional aspects only presented orally were not included. From our experience, discussions in class were often more direct in addressing good scientific practice and work culture. Feedback from teachers often highlighted methodological reflections and the importance of separating normative evaluation from the description. The course was designed as a pass-fail course without specific grades. This setup could have influenced students’ performance either by allowing more freedom and self-direction or by limiting motivation and effort. Additionally, most students took the IBL course as one of the last assignments of the master’s program, often parallel to starting the master’s thesis. This, too, could have influenced motivation and performance.

## Conclusion

Our study has shown, that the use of participant observation is not restricted to learning a scientific practice in a narrow sense. It can also provide students a better understanding of organizational culture and hierarchies of potential future work places within and beyond an academic career in health care. Participant observation is a flexible research strategy which is highly adaptable to (changing) research objectives and field settings – within IBL it is also adaptable concerning comprehensiveness of the learning task. As internships are often an inherent part of degree programs in health sciences, given qualified methodological guidance, similar courses could be implemented in other educational programs.

## Electronic supplementary material

Below is the link to the electronic supplementary material.

Supplementary Material 1

Supplementary Material 2

## Data Availability

The data that support the findings of this study are available from the corresponding author upon reasonable request.

## Abbreviations

CP: Credit Points within the ECTS, 1 CP = 30h workload
ECTS: European Credit Transfer and Accumulation System
IBL: Inquiry-based learning
