# Supplementary material for: Participant observation for inquiry-based learning: a document analysis of exam papers from an internship-course for master’s students in health services research in Germany
Source: BMC Med Educ. 2024 Sep 23;24:1033. doi: 10.1186/s12909-024-05740-4 (PMC11421123; doi:10.1186/s12909-024-05740-4)
Supplement: Supplementary file 1 — Supplementary Material 1 [file 12909_2024_5740_MOESM1_ESM.docx]

**Appendix 1**

**Checklist IBL-Assignment on Participant Observation within an Internship Course**

Master’s Program in Health Service Research and Implementation Science, Heidelberg University, Germany

**I. Presentation**

A 10-15 min presentation on internship experience and observation practice is part of the assignment. The presentation should focus on the selected research experience, first findings and reflection. The presentation should be facilitated by 8-12 slides.

| **A. First slide** |  |
| --- | --- |
| Name |  |
| Topic |  |
| Supervisor |  |
| Date(s) of consultation with supervisor (at least once, before or during internship) |  |
| **B. Research Objective (1-3 slides)** |  |
| Content-related background | What is known so far about the problem?  e.g. specific challenges in healthcare, scientific rigor, organizational and work culture  Provide 2-5 references |
| Theoretical background/concepts | How can the problem be conceptionalized?  e.g. communication theory, management theory  Provide 2-5 references |
| Research focus/research question | Explorative, open, might change over time (Attention: Beware of evaluative and normative questions!) |
| **C. Work Placement (1-2 slides)** |  |
| Characteristics of the employing organization | e.g., sector/industry, size, location, organizational structure, role within the health care system and characteristics of the team/department where the internship took place |
| Main internship activities | e.g., provision of health care, administrative tasks, research, teaching |
| Internship length and dates |  |
| **4. Data Collection (1-2 slides)** |  |
| Context | How, where and when was the data collected? |
| Format | e.g. face-to-face, virtual, telephone |
| Observer involvement | e.g. participant, non-participant observation |
| Observation type | e.g. auto-ethnography, open (with information of the study subjects), covert (to be avoided) |
| References | Provide 1-3 methodological references that were consulted in designing data collection |
| **D. Data Analysis (1-2 slides)** |  |
| Type of data analysis | e.g., content analysis, thematic analysis, GT |
| Steps of data analysis | e.g., familiarization, open coding, focus coding, inductive or deductive coding, memos |
| References | Provide 1-3 reference that were used for designing data analysis |
| **E. Excerpt of field protocols** |  |
| Excerpts of field protocols | Provide 2-3 excerpts of different field protocols with description of the observation, own reflections (analytical notes, see below) and codes/important findings. Excerpts should fit onto one slide |
| **F. Reflection (1-2 slides)** |  |
| Results/findings  a) the observed workplace and participants involved  b) patient care and/or team cooperation  c) health services research | What are the findings concerning the observed work-place?  e.g., conditions, factors explaining first findings (with reference to background/concepts) |
| Lessons learnt | What did I learn from the IBL/internship experience (personally, professionally and work-place related)?  What are my recommendations for students looking for an internship? |
| Methodological reflections, especially concerning:  a) field access  b) participants’ consent  c) observer role | What proposed a challenge during the assignment? How did I solve problems? How did I address research ethics? |
| **G. Optional last slide** |  |
| Questions for discussion |  |

**II. Field Protocols**

Three digitalized field protocols are part of the assignment. Field protocols are based on *field notes*, (often hand-) written within the situation observed. Immediately after observation field notes should be finished adding missing information (timeframe, position of the people involved) as well as questions, impressions, feelings and first interpretations. At home a, a *field protocol* is written on the basis of field notes detailing events, so that they become comprehensible for a non-involved reader. Within the field protocol, a three-column chart is recommended to be used, to differentiate perception-based description from analytical notes and developing themes.

| **A. Observation situation** |  |
| --- | --- |
| Date, time, duration |  |
| Place |  |
| Participants |  |
| Role/involvement of the observer |  |
| Use of sketches or photographs (optional) |  |
| **B. Description of observation (first column)** |  |
| Description of room/setting |  |
| Description of observed participants |  |
| Chronological description of events |  |
| Description of interaction and behaviour |  |
| Description on communication and communication styles |  |
| Description of para- and nonverbal communication |  |
| Direct quotes |  |
| Subheadings, structuring the observation (optional) |  |
| **C. Analytical notes (second column)** |  |
| Documenting inner reactions of the observer, affects, thoughts, questions, interpretations and assumptions of the researcher |  |
| Additional information, answers to questions that came up (gathered afterwards from participants or other sources) |  |
| Reflections of the own role as observer and its possible effect on the situation/observation |  |
| Reference to methodological and content-related literature (optional) |  |
| **D. Emerging themes (third column)** |  |
| Themes based on open coding |  |
| Reference to background literature (optional) |  |
| Reference to conceptional/theoretical literature (optional) |  |
| **E. Pseudonymization** |  |
| Pseudonymization of participant names and data |  |
| Masking of places, time etc. (if deemed necessary) |  |
